# Monitoring diversity in genome-wide association studies requires measuring and reporting on immigration-related factors

**DOI:** 10.3389/fgene.2025.1725866

**Published:** 2026-01-05

**Authors:** Yao Tu, Lindsay Fernandez-Rhodes

**Affiliations:** 1 Department of Biobehavioral Health, College of Health and Human Development, Pennsylvania State University, University Park, PA, United States; 2 Department of Epidemiology, University of North Carolina at Chapel Hill, Gillings School of Global Public Health, Chapel Hill, NC, United States

**Keywords:** Country of birth, country of recruitment, diversity and inclusion, environment, gene-environment (G-E) interaction, genome-wide association studies, immigration

## Abstract

Genome-wide association studies (GWAS) have made remarkable progress to date in deciphering the genetic foundations of complex traits, yet persistent gaps remain in how sample heterogeneity is measured and reported. Current practices typically emphasize diversity by broad ancestry categories or stratification by country of recruitment, but these dimensions alone fail to capture the immigration-related factors that contribute to the genetic or environmental origins of heterogeneity. We argue that incorporating variables, such as country of origin, in descriptions and analyses provides essential context for interpreting genetic associations, particularly in increasingly multi-population and trans-national GWAS samples. We highlight how neglected these variables are in the literature using the GWAS Catalog. We provide suggestions for reporting on these data in future studies. By advocating for a more comprehensive view of diversity in GWAS, we aim to address the under-representation of immigrants in GWAS and thereby strengthen the validity and interpretability of future genomic studies.

## Introduction

Genome-wide association studies (GWAS) play an important role in revealing variants that are associated with specific traits from complex genomic structures. Careful attention to study design and methodology, such as harmonizing data, adjusting or stratifying analyses by key covariates, and incorporating environmental interactors, can substantially improve the discovery and generalization of GWAS findings (Visscher et al., 2017). However, unexplained heterogeneity in the results of these studies can arise from differences in phenotype definitions, environmental exposures, and technical platforms, all of which influence the comparability of results across studies. Monitoring and correcting contributors to heterogeneity has become increasingly important with the growing size of GWAS samples and the increased use of advanced meta-analytic techniques, which enhance study power by combining GWAS summary statistics from across studies and populations (Kulminski et al., 2016; Woodward et al., 2022).

Potential sources of heterogeneity are not limited to genetic factors; non-genetic factors play a major role, as well. Immigration—the voluntary or involuntary movement of individuals across country borders—is ushering in structural changes to an individual’s environmental exposures, behavioral or cultural changes in lifestyles, socioeconomic contexts, etc., that could introduce phenotypic heterogeneity in GWAS (Peterson et al., 2019; Suarez-Pajes et al., 2021). For example, a wealth of research on immigrant populations shows that the prevalence of many diseases, such as obesity, diabetes and hypertension, increases along with the duration of time an immigrant lives in the United States (US) (Oza-Frank et al., 2011; Parikh, 2011; Commodore-Mensah et al., 2016). Describing and modeling such non-genetic sources is therefore essential to the validity of GWAS findings, as it improves our ability to distinguish statistical artifacts from true genetic associations, and provides benchmarks for us to use when examining the external validity of the results to diverse and dynamic populations like immigrants.

### Heterogeneity in GWAS by country context and other immigration-related factors

In GWAS, covariates are used to adjust for potential heterogeneity in the phenotype, and most analyses include standard covariates such as age, sex and principal components, as well as any that are phenotype specific. Inclusion of principal components (or some other global measures of genetic ancestry) can avoid false positive results due to population stratification. Furthermore, the race/ethnicity of participants is usually obtained via self-report, or when missing, inferred by similarity in principal component space, and in some cases may have been used for further batch assignment or stratification. Lastly, although often not acknowledged explicitly, practically speaking, most researchers stratify their analyses by country of recruitment, due to the heterogeneity that separate funding streams, regulations, or research teams impose on the collection of samples. For example, the Health and Retirement Study (HRS) is joined by an international family of 17 studies in different countries across the globe (Lee et al., 2021). Those sister studies are run by different teams in each country and follow similar protocols to the HRS. This research infrastructure is an invaluable resource and could be leveraged to construct thoughtful contrasts that would estimate the heterogeneity caused by different countries of recruitment, above and beyond differences in ancestry. However, their results may still be affected by differences in the countries of origin of their sampled participants, especially in cases where a large share of participants in the sample were born in another country than the country of recruitment.

There are clear disparities in which countries have been able to assemble large enough samples to conduct GWAS, to date. High-income countries, with about 17% of the world population (World Bank, 2021), contributed 84% of GWAS participants, according to data from the GWAS Catalog (Sollis et al., 2022). Low and middle-income countries have contributed a far smaller proportion of GWAS and samples. Most high-income countries also have a high immigration rate from middle-income countries (Spooner et al., 2022), which begs the question of whether GWAS from these countries (especially population representative studies) adequately represent immigrants living within their borders or account for what may be heterogeneity due to immigration-related factors. Thus, we posit that there is added value in incorporating information on immigration and related social factors into GWAS design and analysis.

**FIGURE 1:**
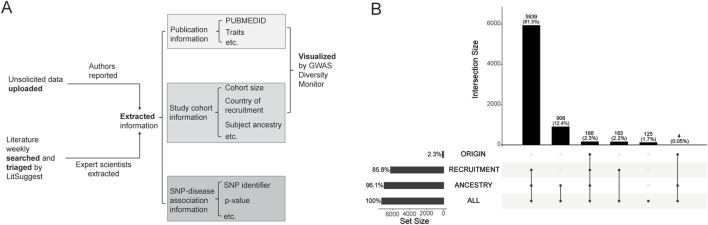
Summary of GWAS Catalog **(A)** Curation Process and **(B)** Genome-Wide Association Studies with Population Descriptors. Panel **(A)** shows a flowchart of the curation process was summarized based on the method descriptions in the GWAS Catalog. Panel **(B)** presents estimates of study numbers that were obtained using the “All ancestry data v1.0” in the GWAS Catalog (7/10/2025), by summing the number of studies with population descriptors (“Broad Ancestral Category”, labeled as “ANCESTRY”; Country of Recruitment, labeled as ‘RECRUITMENT’; and “Country of Origin”, labeled as “ORIGIN”). We filtered out records with identical information on the study’s PubMed ID, stage, ancestry, and country information (country of recruitment and country of origin). The upset plot was plotted by the R package UpSetR (Conway et al., 2017).

As the largest resource of GWAS summary statistics, the GWAS catalog has a well-developed curation process, including both manual and automatic data extraction and cleaning (GWAS Catalog, 2016). Eligible papers are identified through weekly automatic searches using LitSuggest (Allot et al., 2021). Then, full genome-wide GWAS statistics and complementary information are either reported by the author upload (optional) or manually extracted by expert scientists from the literature (Figure 1A). The GWAS Catalog uses ‘broad’ ancestral categories (either explicitly defined or inferred when information is lacking) to describe specific GWAS and the field’s representation in terms of genetic ancestry (Morales et al., 2018). Data on this variable is rarely missing at a study level, as 96.1% of the 7,303 GWAS studies in the GWAS Catalog as of July 2025 reported information on the ancestry of the sample (Figure 1B). Similarly, 85.8% of all cataloged studies had ‘country of recruitment’ information, and when country of recruitment is present for a study, it is very common (97.4%) to also have ancestry information.

**FIGURE 2:**
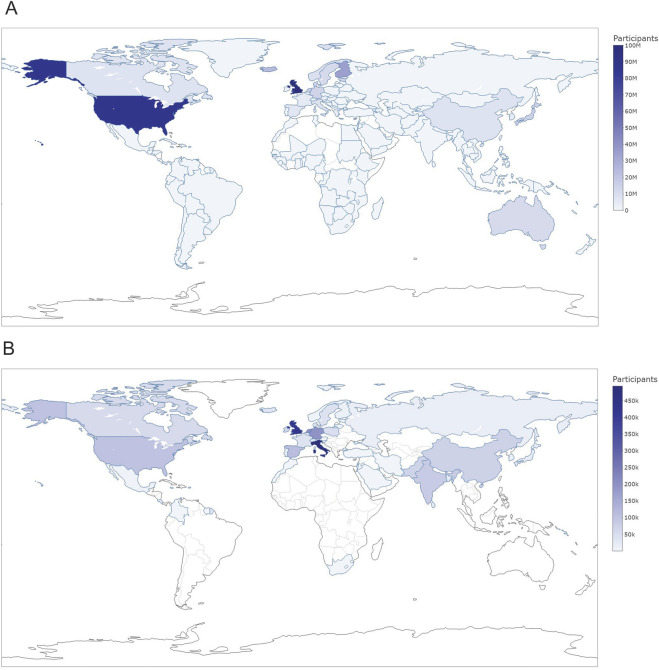
Total Participant Sample in Genome-Wide Association Studies of the GWAS Catalog Mapped by **(A)** Country of Recruitment and **(B)** Country of Origin. Estimates of the total participants per country were obtained by summing the number of individuals across all studies, stages and ancestral categories in the same filtered dataset used in **(B)**. When studies listed multiple countries, we assumed that the number of individuals in this specific study from each country was uniform and calculated the country-specific sample as the number of individuals/number of countries listed. Interactive hyml version of these figures can be founded online: **(A)** https://yao876.github.io/GWAS-Catalog-maps/Participants%20by%20Country%20of%20Recruitment_max100M.html **(B)**: https://yao876.github.io/GWAS-Catalog-maps/Participants%20by%20Country%20of%20Origin.html); if possible, as they are interactive for the user.

Using country of recruitment information downloaded from the GWAS Catalog, the GWAS Diversity Monitor maps out country of recruitment by publication year across all studies, traits/outcomes, and stages (Mills and Rahal, 2020). Following their methods, we have plotted out the distribution of countries of recruitment in the GWAS Catalog since 2008 (Figure 2A). Whenever multiple countries are reported for one publication, we make the strong assumption that they each had equal sample size, which may make the geographic distributions of GWAS samples to date appear more equitable than they really are. Furthermore, whenever the same samples could be included in multiple studies or meta-analyses, the estimated contribution from that country may also be overestimated, which is a limitation of current practices in monitoring diversity in GWAS.

GWAS literature’s current focus on describing and monitoring ‘broad’ ancestral categories and country of recruitment as main indicators of GWAS diversity fails to address more nuanced aspects of ancestral and geographic diversity. Additionally, questions remain about how much residual heterogeneity exists due to unmeasured immigration-related factors, such as the life course exposures that could serve as gene-environment interactors or drivers of phenotypic variability (Fernández-Rhodes, 2023). While most GWAS include age and sex as covariates that may, at least in part, characterize varied immigration waves (e.g., as in the case of younger male guest workers), these covariates do not account for all aspects of the immigration experience. Such additional factors may include the experience of structural inclusionary/exclusionary practices towards certain countries of origin groups, socioeconomic differentials between sending versus host countries, an individual’s reasons for emigration (voluntary emigration versus involuntary displacement) or others.

### Reporting immigration-related factors in GWAS

Although some immigration-related factors (e.g., time living in the US, age at first immigration, immigrant generation, reasons for or timing of most recent immigration) may be unavailable in most datasets, ‘country of origin’ is an easily measured demographic variable that we believe may capture some of the residual heterogeneity that not explained by country of recruitment, ancestry or other standard covariates alone. To answer the question of “What would it take to improve our monitoring of immigrant samples in the GWAS literature?”, we turn again to the GWAS Catalog. Of all GWAS published through July 2025, it becomes clear that remarkably few studies (2.3%) report enough information for a ‘country of origin’ designation. Whenever country of origin is provided in a GWAS publication, information on ancestry is always present (Figure 1B).

This sparse reporting of country of origin is likely due to how the GWAS Catalog defines this variable—the country of origin of the participants’ grandparents, or if lacking, the country of known genealogy (Morales et al., 2018). This definition is in contrast with the way that immigration scholars define country of origin—as being primarily related to the participant themselves. Likewise, a search for country of origin in the PhenX Toolkit suggests a demographic variable for ‘birthplace’ of the participant (Krzyzanowski et al., 2023). In cases where ‘country of birth’ is reported for just the participant or their parents, but there is no specific mention of grandparents or genealogy, based on this definition the GWAS Catalog curators would erroneously list country of origin as not reported. An example of this comes from our own work in the Hispanic Community Health Study/Study of Latinos, where participants’ country of origin, nativity, and Hispanic/Latino background variables, or some combination of them, are routinely reported in the descriptives; of the 31 GWAS published from this study and reviewed separately (Rao et al., 2025), only one had country of origin information in the GWAS Catalog (Burkart et al., 2018). Although the curators may choose to provide an additional ancestry description with supplemental information, it does not appear to be consistently done.

Assuming information on immigration-related factors like a participant’s country of origin exists in most genomic datasets, we recommend two short-term solutions to advance the monitoring of representation of immigrants and improve estimations of them as a source of heterogeneity in genetic studies. First, the GWAS Catalog should prospectively update its definition of country of origin to also include both participants’ and grandparental/genealogic information. Alternatively, an additional variable called ‘participant country of birth’ could be generated for all future data extractions to accomplish the goal of being able to track more information on the diversity and inclusion of the field. Either of these options would have the advantage of better distinguishing between ancestry, grandparental/genealogic origins and a participant’s own country of origin, which in many cases can be different within an individual, and can vary within and across country of recruitment.

Second, the GWAS Diversity Monitor should be redesigned to include an additional map of country of origin, just as they have done for country of recruitment (Figure 2A). Figure 2B shows the dearth of information on country of origin in the GWAS Catalog since 2008, with both a lower total participant sample overall (as compared to Figure 2A) and a different geographic distribution of common countries of origin. The United Kingdom (UK) was one of the most commonly identified countries for both recruitment (largest) and origin (second largest). The UK Biobank has participated in thousands of GWAS publications to date (UK BioBank, 2020), and is commonly in studies with country of recruitment, but not with country of origin information. In contrast, the US is the second most commonly mentioned country of recruitment (Figure 2A), but is not as common as a country of origin (sixth most common, Figure 2B). Italy was among the top countries of recruitment (the thirteen most common, Figure 2A), but surprisingly was the most common country of origin in the GWAS Catalog (Figure 2B). We hope that adding country of origin as an additional visual depiction to the GWAS Diversity Monitor will serve as a reminder for future researchers interested in the diversity of the field to strive to describe immigration-related variables in their own work.

## Discussion

Recent commentaries on the topic of diversity in GWAS have shed light on how immigration-related factors should be considered as another aspect of diversity (Fernández-Rhodes, 2023; Riehm et al., 2023). As described above, we have advanced this work by identifying several challenges to monitoring the inclusion of immigrants in GWAS. We call for future studies, and particularly new data-generating initiatives, to measure several immigration-related factors in their participants using established methods such as those in the PhenX Toolkit (Krzyzanowski et al., 2023) and report on these factors in the GWAS descriptives, just as they would do for age, sex, etc.

Immigration-related factors may include country of birth of the participant, birthplace of parents or grandparents, years of residence, or even go beyond these measures to include variables like age at first immigration, immigrant generation, reasons for migration (forced versus voluntary), patterns of seasonal migration, and current citizenship or legal immigration status, provided that the privacy of this information can be protected. Calls have been made for the inclusion of these variables and other variables in US-based national surveys (Marouf et al., 2025) as well as in studies outside of the US context (Pathak et al., 2021). We recognize that not all datasets have information on such immigration-related factors, and even when it is collected, the data may not be missing at random and should be analyzed with this in mind.

Nonetheless, a greater availability of such data in the larger genomics ecosystem should allow researchers to better describe and possibly also account for the potential heterogeneity related to immigration-related factors. As genomics researchers begin to work with more immigration-related variables, they should have the ethical, legal and social implications of their work always in front of mind. Whenever possible, investigators should foster community-engaged and informed designs, as a means of ensuring that communities are able to actively guide the manner in which immigration-related variables are incorporated in GWAS descriptives and analyses (Lemke et al., 2022; Ravi et al., 2025; Valdez-Aguilar et al., 2025). Yet, numerous challenges remain to ensuring that all individuals and communities, immigrant and non-immigrant, feel that their privacy is adequately protected amidst changing political times.

For example, in the US research context, crowd-sourced resources estimate that more than several hundred research projects have been terminated by the National Institutes of Health (NIH) and National Science Foundation in the first months of 2025 (Kozlov, 2025; Kozlov and Ryan, 2025; Ross et al., 2021), totaling at least $4 billion dollars of lost research support (Nienaber and Leeming, 2025). Although the exact methodology that was used to flag grants remains unclear, most of these grants were focused on diversity, equity and inclusion, health disparities, and included either investigators or participants from under-represented groups in genomics (Kozlov and Ryan, 2025; Nienaber and Leeming, 2025). Simultaneously, the NIH has seen a 6% reduction in force, delays in grant reviews, and all-time low success rates (Kozlov, 2025). In the 2025 fiscal year, the NIH funded several thousand less grants than in the previous fiscal year, by multi-year disbursements to fewer awardees in the final months of the fiscal year.

In light of long-standing disparities in the participation of women and minorities in NIH-funded research that predate such recent developments [for example, (NIH, 2023)], it may become harder to engage under-represented, marginalized groups, such as immigrants, in genomic studies in the near future. Scholars have expressed concern about the future of NIH-issued Certificates of Confidentiality (NIH, 2024) in protecting the privacy of sensitive participant data in both genomics and other areas of health research (Wolf et al., 2024; Kozlov, 2025; LoTempio and Moreno, 2025). Thus, it may be important for researchers to consult with legal scholars, bioethicists, or form other interdisciplinary connections to guard against individual harms or discuss what may be population-level sensitivities. Greater uptake of community-engaged initiatives may be one way to better understand immigration as a facet of diversity that has been largely missing in the GWAS literature to date, as well as address the historical under-representation of certain groups in genomics research (Lemke et al., 2022).

Immigration is an important contributor to environmental exposures, behavioral/cultural practices, lifestyles and the socioeconomic circumstances, in which study participants find themselves. Immigration-related variables should be explicitly described and considered as analytic variables in future GWAS and genomics initiatives, including diversity monitoring. By advocating for more comprehensive diversity metrics, we hope that future research can address the under-representation of immigrants in GWAS, while strengthening the validity and interpretability of their genomic findings.

## Data Availability

The datasets presented in this study can be found in online repositories. The names of the repository/repositories and accession number(s) can be found below: We obtained information on published GWAS in the GWAS Catalog file named ‘All ancestry data v1.0’ here: https://www.ebi.ac.uk/gwas/docs/file-downloads, accessed on July 18, 2025.
